# Examining the Effectiveness of Discriminant Function Analysis and Cluster Analysis in Species Identification of Male Field Crickets Based on Their Calling Songs

**DOI:** 10.1371/journal.pone.0075930

**Published:** 2013-09-25

**Authors:** Ranjana Jaiswara, Diptarup Nandi, Rohini Balakrishnan

**Affiliations:** Centre for Ecological Sciences, Indian Institute of Science, Bangalore, Karnataka, India; Aberystwyth University, United Kingdom

## Abstract

Traditional taxonomy based on morphology has often failed in accurate species identification owing to the occurrence of cryptic species, which are reproductively isolated but morphologically identical. Molecular data have thus been used to complement morphology in species identification. The sexual advertisement calls in several groups of acoustically communicating animals are species-specific and can thus complement molecular data as non-invasive tools for identification. Several statistical tools and automated identifier algorithms have been used to investigate the efficiency of acoustic signals in species identification. Despite a plethora of such methods, there is a general lack of knowledge regarding the appropriate usage of these methods in specific taxa. In this study, we investigated the performance of two commonly used statistical methods, discriminant function analysis (DFA) and cluster analysis, in identification and classification based on acoustic signals of field cricket species belonging to the subfamily Gryllinae. Using a comparative approach we evaluated the optimal number of species and calling song characteristics for both the methods that lead to most accurate classification and identification. The accuracy of classification using DFA was high and was not affected by the number of taxa used. However, a constraint in using discriminant function analysis is the need for a priori classification of songs. Accuracy of classification using cluster analysis, which does not require a priori knowledge, was maximum for 6–7 taxa and decreased significantly when more than ten taxa were analysed together. We also investigated the efficacy of two novel derived acoustic features in improving the accuracy of identification. Our results show that DFA is a reliable statistical tool for species identification using acoustic signals. Our results also show that cluster analysis of acoustic signals in crickets works effectively for species classification and identification.

## Introduction

Traditional taxonomy, which involves discovering and identifying new species using key morphological characters and matching them with the characters of voucher specimens, has contributed to biodiversity exploration since the time of Linnaeus. However, the process of biodiversity exploration and estimation slows down in the tropics due to high species diversity, lack of sufficient numbers of active trained taxonomists [1] and also often due to the inaccessibility of the holotype specimens that are necessary for confirming species identity.

This problem can be even more confounding in the case of arthropods which have enormous diversity, especially in the tropics [1]. Several attempts have been made to use technological advances in the fields of molecular biology and engineering to overcome this problem. In this modern era of taxonomy, many new methods for systematic study of organisms are in use such as DNA barcoding [2], [3] and Web-based taxonomy [4]–[6]. Traditional taxonomy based on morphology fails to identify taxa that appear morphologically very similar to each other and in such cases, molecular taxonomy using DNA sequences becomes one of the important approaches in identification of cryptic species [7].

Advertisement calls, which are produced in various behavioral contexts in many taxa such as birds, frogs, cicadas and orthopterans [8] help in pre-mating isolation [9]–[11] between species and can thus be useful in systematics [12]. Acoustic signals have mostly been used as an additional tool in identification of cryptic species [13]–[15] along with molecular and morphological characters [16]. Differences in the calling songs of Hawaiian crickets of the genus Laupala have been shown to correspond to species boundaries obtained on the basis of mitochondrial DNA variation [17], [18]. Therefore, advertisement calls, because of their species-specificity, can be used to complement DNA sequence data. Concordance in the results obtained from the cluster analysis of calling songs and clusters based on morphological characters has illustrated the ability of acoustic features to delimit species boundaries in four sympatric species of tree crickets of the genus Oecanthus [19]. Thus, species-specific calling songs can be used as a reliable feature for taxonomic identification and classification [20], [21].

Due to the inaccessibility of the reference specimens and taxonomic literature, which are largely available in North American and European museums, new methods have been developed such as automatic identification of species based on their morphological characters [22] or acoustic signals [23]–[25]. The “Orthoptera Species File Online” is a Web-based catalogue developed for the insect Order Orthoptera where different kinds of information such as classification, distribution, pictures of holotype specimens and references to background literature have been incorporated. Despite this development, the available information on crickets is still not sufficient to make species-level identification. For many of the holotype specimens, images are absent and at times key taxonomic characters are not present. Morphological identification of crickets to the species level is thus difficult but given the distinct and species-specific structures of their calling songs, these may be employed together with distribution information to identify species in a rapid, reliable and non-invasive manner.

Identification based on morphological characters requires collection of animals and involves extensive surveys in the field whereas identification based on acoustic signals does not require collection of specimens. Acoustic signals of animals can be recorded easily and the recordings can be used for species identification either by analyzing the acoustic features using statistical methods or using these features in training algorithms for their automatic identification. This is documented in many studies using linear discriminant analysis [26], [27], decision trees [28], artificial neural networks [29]–[32], hidden Markov chains [33] and support vector machines [25]. All of these algorithms perform well in automatic identification with an accuracy of >90% [34]. There exist a large number of studies in which acoustics has been used independently for species identification in bats [27], [35]–[39], fish [26], [40]–[46], birds [47], [48], frogs [49]–[53], crickets [23] and dolphins [54]. Most of the studies listed above have used discriminant analysis for the classification of organisms into different groups. Some of the recent studies have also used an automated identifier based on their calls [23], [32]. Discriminant function analysis, regardless of its requirement for a priori definition of groups is powerful since the percentage of correct classification is 80–96% [27], [35], [38].

Therefore, in this study we used discriminant function analysis (DFA) to evaluate its strength in species identification using calling songs of field crickets belonging to the subfamily Gryllinae. We also aimed to compare the efficacy of DFA with cluster analysis. As discussed in the above sections, in case of cluster analysis the accuracy in classification was almost 100% when used for only four species of crickets. The percentage of accurate classification is however unknown when these methods are employed for higher numbers of species. Therefore, we used two methods in this study, discriminant function analysis (DFA) and cluster analysis. We examined the influence of varying the number of call types on their efficiency in classification.

We addressed the following questions: What is the optimum number of species to be used in the statistical analysis of acoustic signal features to obtain a correct classification? How many and which acoustic characters should be used in such an analysis? Is there any relation between number of taxa and characters to achieve clear and well resolved groups of individuals reflecting species? We also compared the power of two statistical methods i.e. discriminant function analysis and cluster analysis in correctly assigning species based on their acoustic signals.

## Materials and Methods

### Ethics statement

Necessary permits for all locations sampled in this study were obtained from the National Biodiversity Authority, Government of India. None of the species in this study are listed as endangered and were not collected from protected areas.

### Sampling area and studied material

**Figure 1:**
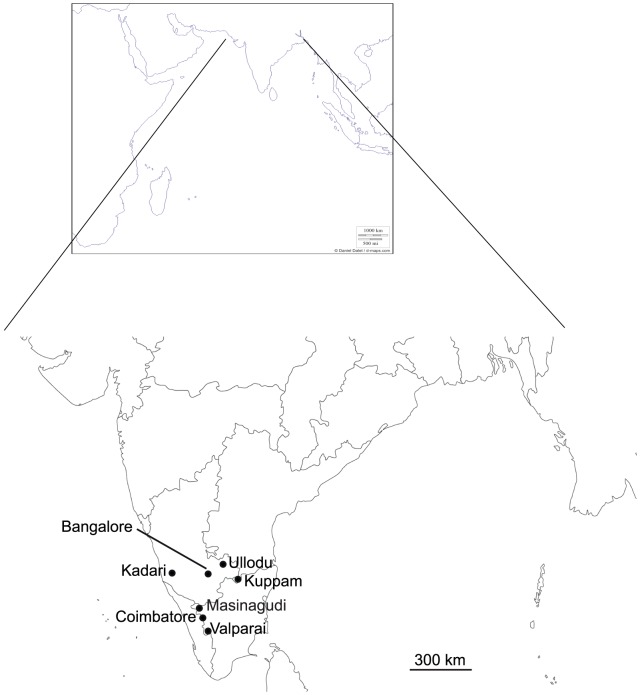
The sampling sites of field crickets in Southern India.

Extensive sampling of field crickets was performed to maximize the possibility of capturing different call types which were distributed in and around Bangalore within a range of 500 km in the southern parts of India. Coimbatore (11.09°N, 76.78°E), Kadari (13.2°N, 75°E), Valparai (10.32°N, 76.95°E), Masinagudi (11.57°N, 76.64°E), Kuppam (12.82°N, 78.25°E) and Ullodu (13.64°N, 77.7°E) were the sampling sites (Fig. 1). Calling songs of field cricket species were recorded and individuals were collected and preserved in 70% ethanol (Figures S1, S2, S3, S4, and S5, Table 1) for morphological identification. The specimens are stored in the Centre for Ecological Sciences, Indian Institute of Science, Bangalore, India.

**Table 1:** Mean (standard error) values of song features of the 14 species of field crickets used for the study.

Genus	Collection site	Call period (ms)	Call duration (ms)	Syllable period (ms)	Syllable duration (ms)	Dominant frequency (kHz)	Relative variance (ms)	Constancy factor (ms)	No. of individuals
*Coiblemmus*	Coimbatore	93.2 (1.9)	37.3 (1.4)	23.8 (1.3)	14.1 (0.9)	5.3 (0.1)	943.2 (22.4)	843.9 (29.9)	5
*Gryllodes*	Kuppam	130.1 (6.4)	46.8 (1.6)	16.3 (0.7)	20.6 (0.6)	5.9 (0.1)	59.7 (8)	5.6 (0.9)	6
*Gryllus*	Bangalore	345.5 (11.9)	166.9 (4.3)	36.8 (1.3)	20.6 (0.6)	5 (0.1)	57 (9.4)	16.9 (2.6)	6
*Itaropsis* subspecies 1	Bangalore	11342.9 (915.5)	8343.1 (983.2)	99.7 (2.8)	40 (1.1)	6.6 (0.2)	5.5 (1.4)	7.5 (1)	6
*Itaropsis* 2	Kadari	1155.6 (85)	420.6 (76.8)	42.5 (2.3)	22.3 (1.2)	7.5 (0.1)	67.7 (10.4)	67.4 (8.8)	5
*Itaropsis* 3	Valparai	1504.4 (122.2)	296.5 (16.8)	54.9 (1.3)	26.4 (0.8)	6.5 (0.1)	125.1 (18.7)	122.5 (17.2)	7
*Phonarellus* sp.1	Kadari	467 (14.6)	312.1 (22.2)	35.8 (1.8)	12.8 (0.9)	6.9 (0.1)	124.7 (25.6)	35.5 (5.1)	6
*Phonarellus*	Bangalore	68.9 (1)	36.2 (0.6)	34.5 (0.5)	13.8 (0.2)	6.6 (0.1)	30.9 (2.8)	2.3 (0.2)	6
*Platygryllus*	Mudumalai	667.5 (43.2)	317.5 (15.6)	26.6 (0.5)	16.3 (0.4)	4.4 (0.1)	118.8 (14.2)	64.5 (6.7)	8
*Plebiogryllus*	Ullodu	217.8 (7.7)	122.7 (6.4)	31 (0.6)	17.7 (0.4)	6 (0.1)	224.8 (36.7)	57.4 (13.3)	7
*Teleogryllus*	Kadari	305.9 (13.7)	115.7 (12.2)	54.5 (1.2)	41.2 (0.9)	3.1 (0.1)	211.5 (17)	64.2 (5.8)	6
*Turanogryllus*	Mudumalai	863.2 (29.7)	600.6 (34.2)	8.9 (0.2)	5.6 (0.3)	6.2 (0.1)	167.2 (16.5)	136.2 (18.7)	8
*Velarifictorus* sp.1	Kuppam	9271 (1445.2)	1090.4 (136.8)	42.3 (1.8)	24 (0.8)	4.4 (0.1)	541.5 (268.4)	1692.7 (330.9)	5
*Velarifictorus* sp.2	Kadari	318.6 (26.4)	144.4 (17.6)	38.6 (1.4)	24.2 (0.8)	4.2 (0.0)	251.2 (42.9)	1474.4 (101.3)	4

### Song recordings and analysis

**Figure 2:**
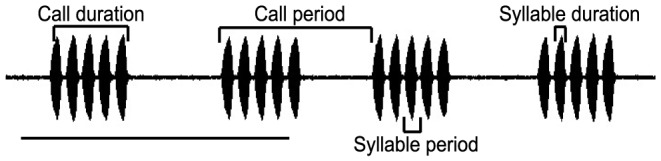
Oscillogram of *Gryllus bimaculatus* illustrating measured temporal features. Scale bar represents 0.5 second.

Individual males were located in the field by listening to their calling songs and tracking them by ear. After visual confirmation of the calling male's position, its calling song was recorded using a Sony WM-D6C Professional Walkman cassette recorder and a Sony ECM-MS957 microphone (flat frequency response from 50 Hz to 18 kHz) with the microphone at a distance of 15 cm from the male. Ambient temperature was measured close to the calling male using a Kestrel 3000 Pocket Weather Station. The same male was captured and preserved in 70% ethanol for further studies. The recorded calling songs of 85 individual field crickets (4–8 individuals per species) were digitized using a Creative Sound Blaster A/D Card at a sampling rate of 44.1 kHz for spectral and temporal analysis. Spectral analysis of the digitized signal was carried out using the signal processing software Spectra Plus Professional (1994, Version 3.0, Pioneer Hill Software, Poulsbo, WA, USA). Spectral analysis was performed on recorded calling songs of duration 1–1.5 minutes with the exception of the genus Coiblemmus where 5.6 minutes of recorded call was used for the analysis. Dominant frequency of the recorded calling song was measured by generating a power spectrum using a Fast Fourier Transform (FFT) with a Hamming window and a window length of 2048 sampling points. As the calling songs of field crickets are of narrow bandwidth, the dominant frequency i.e. frequency with maximum energy is represented by a narrow peak with highest amplitude. Temporal pattern analysis was performed using a custom-built program (Chandra Sekhar, EE, IISc) in Matlab (2001, Version 6.1.0.450, The Mathworks Inc., Natick, MA, USA). Syllable duration, syllable period, call duration and call period (Fig. 2) were measured as the key features of the temporal pattern. In case of trilling call types, call duration was measured by considering the number of bouts present within the recorded call of 1–1.15 min duration. Crickets are poikilothermic animals and thus several of the calling song features vary with change in the environmental temperature. As the calling songs of the field crickets were recorded from different localities, the temperature of song recordings varied from 21–28°C. All the different acoustic features were initially regressed with their corresponding temperature recordings. If the regression was found to be significant, then using the linear regression equation for the particular acoustic feature, the corresponding values of the acoustic feature were calculated at 25°C.

Apart from the fine temporal and spectral features, two novel temporal characters were used to resolve the diversity and complexity of acoustic signals. In general, syllables are either arranged in the form of distinct chirps, which repeat in a predictable way or as continuous trills. However, in two species of field crickets, Velarifictorus sp.2 and Coiblemmus sp., we found grouping of chirps. These chirp groups had a repetitive pattern like the chirps themselves. This higher order structure is not captured by features such as chirp period and chirp duration (Fig. 2). Therefore, two novel acoustic features, ‘Constancy factor’ and ‘Relative variance’ were used. Constancy factor was defined as the summation of the modulus of differences between the successive chirp periods normalized by the total number of chirps: 
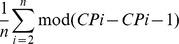



Relative variance [55] was measured as the standard deviation of the natural logarithms of chirp periods. Calling songs with two levels of organization of chirps were expected to have higher values of both constancy factor and relative variance compared to that of the other calling songs.

### Statistical analysis

Two kinds of statistical approaches were employed in this study i.e. discriminant function analysis and cluster analysis in identifying species based on acoustic data. Both of these analyses were performed in Statistica (Statsoft Inc., Tulsa, OK, USA).

As the aim of this study was to determine the optimal number of species that could be used in correct identification, the number of taxa used in each analysis was varied from 5 to 13. The rationale behind choosing the lowest number of taxa as five for the analysis is that it is known from a previous study that the use of four taxa in cluster analysis performed very well in species boundary delimitation based on acoustic features.

Out of the 14 call types recorded from the different sampling sites, 5 to 13 taxa were selected randomly using a random number generator in R version 2.14.1 [56]. There were nine different groups with varying numbers of taxa, ranging from 5 to 13, each with ten replicates. The taxa groups were named based on the number of taxa included in the analyses, for example, the group including nine taxa is referred to as taxa9. To assess the effects of the number of acoustic features on correct classification of taxa, the number of acoustic features was also varied, using five and seven features respectively. The five standard acoustic features included syllable duration, syllable period, call duration, call period and dominant frequency whereas in the case of seven features, two new acoustic features i.e. relative variance and constancy factor were added for the analysis. These two new song features were defined by observing the super-structured temporal pattern of the calling songs of two species of field crickets of the genus Coiblemmus sp. and Velarifictorus sp.2. Thus for each of the two character sets, there were 90 (9 taxa groups ×10 replicates) cases. To evaluate the effect of number of taxa and number of acoustic characters on the classification, the same set of 10 replicates was used for performing both the discriminant function and cluster analysis.

In discriminant function analysis (DFA), all the individuals included in the study are classified into different groups a priori based on some information about the taxa. Therefore, in this study we classified individuals of field crickets into different groups representing different species based on the detailed study of their external and internal morphological characters and using the keys of Chopard [57]. There were also certain cases where some of the species could not be identified to known species using these keys despite their morphological features being specific and distinct. In the case of the genus Itaropsis a single species is known from the Indian subcontinent however, an analysis performed on the combined data set (morphology and molecular data) revealed three subspecies with distinct songs [16]. Two rounds of DFA were performed, first with five and then with seven acoustic features, for all the sets of randomly selected taxa. For DFA, we used groups (defined by classifying individuals with similar morphological characters together) as the dependent variable and the acoustic features as independent variables. A classification matrix was derived for each of the 180 (90×2) different sets. To investigate the effect of number of individuals per taxon on the DFA results, the entire analysis was repeated with a data set containing five individuals per taxon (achieved by removing individuals randomly from the total sample size for each taxon). We also randomly misclassified some individuals of the total data set *a priori* to study the effect of erroneous classification based on morphology. We misclassified 5%, 10% and 20% of the individuals of one particular group with eight randomly selected taxa and seven acoustic features separately. The analysis was iterated ten times for each of the three misclassification sets.

For cluster analysis, measured acoustic features were standardized by subtracting the mean from each value and then dividing by the standard deviation. All the acoustic data were pooled together and pairwise Euclidean distances were calculated. The distance matrix thus obtained summarizes distances between all paired individuals. This matrix was then subjected to cluster analysis (single linkage) to examine groups emerging on the basis of overall call similarity between individuals. This exercise was performed for all the sets of randomly selected taxa and characters to evaluate the efficiency of cluster analysis in grouping individuals correctly into groups reflecting species. In the dendrogram derived from cluster analysis, a linkage distance of 0.4 was used as an objective criterion for defining individuals that were grouped together as belonging to the same species. Thus, there was a total of 180 (2 character sets ×9 taxa groups ×10 replicates) data points with two categories each, number of taxa (with nine levels) and number of characters (with two levels). To investigate the effect of these two categories (taxa groups and character sets) on the proportion of correct classification, a Two-Way Analysis of Variance was conducted using a Generalised Linear Model with binomial family of errors and a Logit link function. All the analyses were carried out in R version 2.14.1. The default contrast settings were used to compare taxa5 with all the other eight taxa groups in a pairwise manner. Contrasts were then changed in a way that would give all the possible pairwise comparisons between taxa8 and the rest of the taxa groups. Bootstrapping was performed in Matlab version 6.5, with 100 iterations for each of the nine taxa groups separately for the two different sets of characters. The binomial data were arc sine transformed. Using the standard errors and the means of the distribution generated by bootstrapping, confidence intervals were calculated. The means and confidence intervals were reverse arc sine transformed to obtain the actual proportions.

The robustness of the results of cluster analysis could depend on the number of replicates as well as the number of individuals per taxon used in the study. To investigate the effect of number of replicates on the results of cluster analysis, five more replicates were added to the initial data set of ten replicates and GLM was carried out with the pooled dataset of fifteen replicates separately. To investigate the effect of sample size, the number of individuals was randomly reduced to five for all the taxa before carrying out cluster analysis.

In order to quantify the effect of two novel acoustic characters, it was important to retain the only two species that had a complex calling song pattern in all the clusters. Thus, more clusters were generated with 6, 7 and 10 taxa. In each of these taxa groups, Velarifictorus sp.2 and Coiblemmus sp. were retained and the rest of the taxa were randomly selected. These randomizations were repeated ten times for each of the 3 taxa groups, with 5 and 7 characters separately. Finally, for each of the two species Velarifictorus sp.2 and Coiblemmus, the number of times these were correctly resolved out of 10 randomizations was calculated. The proportions of successful identification were calculated for clusters with five and seven characters across the 3 different taxa groups. These proportions were compared using a Binomial Test for equality of proportions [58] for the two species separately. Thus for each of the two species there were three pairwise comparisons for the three different taxa groups.

## Results

### Discriminant function analysis

The results of discriminant function analysis performed on all the ten replicates for 5 to 13 taxa are shown in Table 2. The percentage of correctly classified individuals into their predefined group was found to be always 100% for five and six taxa based on both five and seven acoustic characters in all the ten replicates. For seven to ten taxa, the percentage value was 98–100% in all the replications except for five cases where it was found to vary between 95–97%. The percentage classified correctly reduced on further increase in number of taxa i.e. from eleven to thirteen, however it was still almost 95% correct. Overall, the discriminant analysis revealed an increase in the accuracy of classification when the number of acoustic features was increased from five to seven. Reducing the number of individuals per taxon to five by random removal of individuals did not affect the accuracy of classification (Table S1). *A priori* misclassification of individuals yielded accuracies that varied between 80–94%, on average, in case of 20–5% misclassification respectively (Table S2).

**Table 2:** Percentage of correctly allocated individuals by discriminant function analysis (DFA).

Number of taxa and characters	1^st^ randomization	2^nd^ randomization	3^rd^ randomization	4^th^ randomization	5^th^ randomization	6^th^ randomization	7^th^ randomization	8^th^ randomization	9^th^ randomization	10^th^ randomization	Average of correct classification
5 T:7 C	100	100	100	100	100	100	100	100	100	100	100
5 T:5 C	100	100	100	100	100	100	100	100	100	100	100
6 T:7 C	100	100	100	100	100	100	100	100	100	100	100
6 T:5 C	100	100	100	100	100	100	100	100	100	100	100
7 T:7 C	100	100	100	97.8	100	100	100	100	100	100	100
7 T:5 C	100	100	100	91.1	100	100	100	100	100	100	99
8 T:7 C	100	97.8	100	100	100	100	100	100	100	100	100
8 T:5 C	100	91.8	100	100	100	100	100	100	100	100	99
9 T:7 C	94.5	100	100	100	98.2	94.7	100	100	100	100	99
9 T:5 C	92.7	100	100	100	92.7	92.9	100	100	100	100	98
10 T:7 C	100	100	100	100	100	100	100	100	100	100	100
10 T:5 C	100	100	100	93.1	100	100	100	93.3	100	100	99
11 T:7 C	100	98.4	100	98.4	98.3	100	96.8	98.4	98.5	98.4	99
11 T:5 C	100	93.7	100	93.7	93.5	100	93.7	93.7	89.5	93.7	95
12 T:7 C	100	95.9	95.8	95.7	100	98.6	100	91.1	100	95.4	97
12 T:5 C	100	94.6	94.4	94.4	100	94.6	100	91.2	100	93.8	96
13 T:7 C	96.2	96.2	94.9	96.2	96.1	98.7	96.1	96.1	100	100	97
13 T:5 C	94.9	94.9	92.4	95	94.9	95	94.8	94.8	100	100	96

**xT:yC in the row headers refer to ‘x’ taxa with ‘y’ characters used for DFA.**

### Cluster analysis

**Figure 3:**
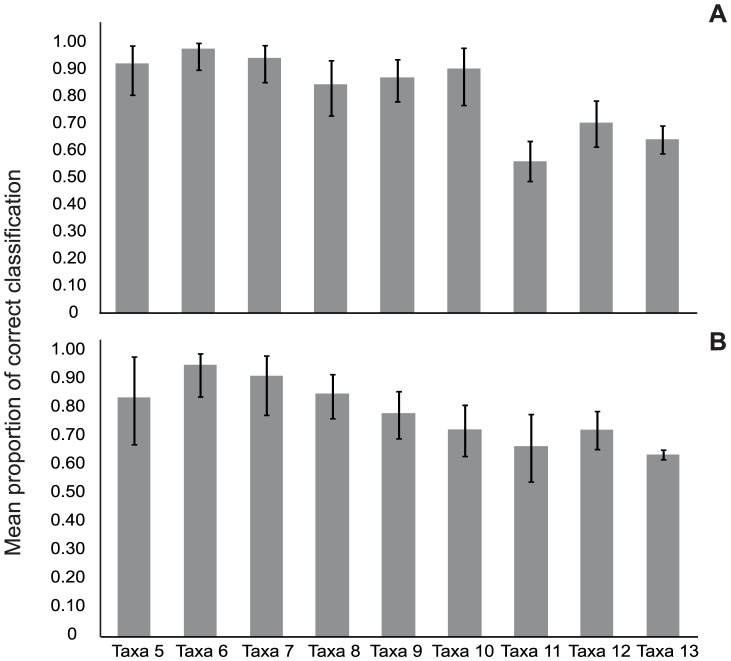
Mean proportion of correct classification as derived from bootstrapping with 100 iterations. Error bars represent 95% confidence interval. (A) Clusters with 5 acoustic characters. (B) Clusters with 7 acoustic characters.

The results of cluster analysis based on five and seven acoustic features for the ten replicates of the number of taxa varying from five to thirteen are shown in Table 3. The percentage of correct clustering of individuals belonging to a species was comparatively lower than that obtained from discriminant analysis. However, accurate classification of individuals into species (85–90%) was obtained in the case of six or seven randomly selected taxa. With the increase in the number of taxa from seven to ten, the accuracy in classification level reduced but was still found to be 82% correct on average. From eleven taxa onwards, the percentage of correct classification reduced to 64%. On bootstrapping, the trend remained similar between the clusters with 5 acoustic traits (Fig. 3A) and those with 7 acoustic traits (Fig. 3B).

**Table 3:** Percentage of correctly allocated individuals by cluster analysis.

Number of taxa and characters	1^st^ randomization	2^nd^ randomization	3^rd^ randomization	4^th^ randomization	5^th^ randomization	6^th^ randomization	7^th^ randomization	8^th^ randomization	9^th^ randomization	10^th^ randomization	Average of correct classification
5T:5C	100	60	60	60	60	100	60	60	100	100	76
5T:7C	100	100	60	80	60	100	80	100	80	100	86
6T:5C	33	100	100	100	67	100	100	100	83	100	88
6T:7C	50	100	100	100	67	100	100	100	83	100	90
7T:5C	100	86	100	71	100	100	100	57	57	86	86
7T:7C	100	86	100	86	100	100	100	71	71	86	90
8T:5C	75	75	75	75	88	100	88	100	75	75	83
8T:7C	75	75	88	88	88	100	63	88	50	100	81
9T:5C	78	56	78	78	56	78	100	67	89	78	76
9T:7C	78	67	100	78	67	78	100	89	89	89	83
10T:5C	90	90	60	60	70	60	90	60	50	80	71
10T:7C	90	90	100	60	100	100	90	60	60	100	85
11T:5C	64	55	64	55	64	100	55	55	64	64	64
11T:7C	45	55	64	36	64	82	55	55	45	55	55
12T:5C	67	75	67	50	75	67	92	67	83	75	72
12:7C	67	58	58	58	67	83	92	83	75	58	70
13T:5C	62	62	69	62	62	62	62	62	69	69	64
13T:7C	62	62	62	46	62	85	62	62	69	69	64

**xT:yC in the row headers refer to ‘x’ taxa with ‘y’ characters used for cluster analysis.**

In the GLM analysis (Table 4), taxa5 was first compared with the rest of the eight taxa groups containing six to thirteen taxa. The proportion of correct classification decreased significantly when more than 10 taxa were used in the cluster analysis (Table 4).

**Table 4:** Results of the Generalised Linear Model analysis with all the pairwise comparisons between taxa5 and the other eight taxa groups and their interactions with character sets.

Categories	z Value	P Value
taxa5 (intercept)	4.454	0.000008
taxa6	0.940	0.347313
taxa7	0.670	0.502782
taxa8	−0.700	0.483626
taxa9	−0.415	0.678184
taxa10	−0.163	0.870386
taxa11	−3.544	**0.000395**
taxa12	−2.134	**0.032859**
taxa13	−2.791	**0.005248**
character2	−1.262	0.207020
taxa6:character2	0.357	0.721072
taxa7:character2	0.346	0.729065
taxa8:character2	1.120	0.262556
taxa9:character2	0.282	0.778217
taxa10:character2	−0.278	0.780797
taxa11:character2	1.691	0.090797
taxa12:character2	1.245	0.213159
taxa13:character2	1.132	0.257484
Residual deviance: 215.35 on 162 degrees of freedom		
AIC: 585.65		

When taxa8 group was compared with all the other taxa groups in a pairwise manner (Table 5), no significant differences were detected in the proportions of correct classification between taxa8 and the other taxa groups with five to ten taxa (Table 5). However, taxa groups with 11 and 13 taxa showed a significant decrease in proportion of correct classification from that of taxa8. There was no significant difference in accuracy of classification between clusters generated using 5 and 7 characters (character2, Table 4 & 5). There were no significant interactions between clusters with different number of taxa and different sets of characters.

**Table 5:** Generalised Linear Model with all the pairwise comparisons between taxa8 and the other eight taxa groups and their interactions with character sets.

Categories	z Value	P value
taxa8 (Intercept)	5.119	**0.0000003**
taxa5	0.700	0.483626
taxa6	1.700	0.089108
taxa7	1.489	0.136357
taxa9	0.355	0.722230
taxa10	0.670	0.503080
taxa11	−3.618	**0.000297**
taxa12	−1.774	0.076023
taxa13	−2.643	**0.008225**
character2	0.205	0.837424
taxa5:character2	−1.120	0.262556
taxa6:character2	−0.618	0.536520
taxa7:character2	−0.735	0.462381
taxa9:character2	−1.017	0.309071
taxa10:character2	−1.698	0.089423
taxa11:character2	0.518	0.604385
taxa12:character2	−0.007	0.994293
taxa13:character2	−0.174	0.862092
Residual deviance: 215.35 on 162 degrees of freedom		
AIC: 585.65		

The proportion of correct classification decreased significantly when more than ten taxa were analyzed together, in both the analyses with fifteen replicates as well as with reduced number of individuals per taxon (Tables S3 & S4 respectively).

### Effect of constancy factor and relative variance on call superstructure resolution

Binomial tests revealed that the proportion of correct resolution increased significantly for Coiblemmus with the introduction of two novel characters (p<.01, Table 6) in all the three taxa groups. However, for Velarifictorus sp.2 the proportions did not change significantly (Table 6).

**Table 6:** Binomial Test results for proportions of correct classification of Velarifictorus sp.2 and Coiblemmus sp.

	*Velarifictorus* sp.2		*Coiblemmus* sp.	
	Chi-squared Estimate	P value	Chi-squared Estimate	P value
6 taxa	0.31	0.58	**7.27**	**0.007**
7 taxa	0.21	0.65	**10.21**	**0.001**
10 taxa	0.81	0.37	**10.21**	**0.001**

## Discussion

### Comparison of DFA and cluster analysis as tools for species classification and identification

In this study, we used song features of field cricket species and subjected them to two different kinds of statistical analysis i.e. discriminant function analysis (DFA) and cluster analysis to examine their efficacy in species identification. DFA was able to classify individuals with an accuracy of 95–100% for up to 13 species considered together. The high accuracy of classification based on DFA was not affected by reducing the number of individuals per taxon. Even with *a priori* misclassification of 20% of the individuals, DFA yielded eighty percent accuracy, implying its robustness as a classification tool. The high accuracy of classification of crickets to the species level using DFA is concordant with previous studies. 79% of echolocating bats belonging to twelve species from Britain were correctly classified based on 13 acoustic variables using DFA [27]. Similar studies have reported overall 80–82% correct classification of 22 bat species from Italy [35] and 8 bat species from Japan [37]. DFA has also been used in identification of birds and frogs [34] and the average of correct classification for both the taxa was found to be 71%. In nine species of dolphins of the Pacific Ocean, the accuracy of classification was found to be 41% using DFA.

In our study, correct classification based on cluster analysis varied from 55–90% with varying number of taxa (5–13). In cluster analysis, accuracy of classification was optimal for six to seven taxa, considered simultaneously, and dropped significantly with more than ten taxa. Cluster analysis is sensitive to the number of individuals per taxon. Even with reduced number of individuals per taxon, accuracy of classification, however, did not differ significantly for up to ten taxa. This pattern was also observed when higher replicate sizes were used in the analysis suggesting greater robustness of our results.

Even though the percentage of correct classification was comparatively low in the case of cluster analysis when compared with DFA, cluster analysis can be more useful in situations where no prior knowledge or basis of grouping all individuals is available. This is frequently the case with tropical insects, where there are a large number of unknown species or those whose acoustic signals have not been recorded, commonly co-existing with known species. Once species have been identified and their call features are known, their song features can be used in training algorithms and species identification can be automated using DFA as a statistical tool. Automated recognition of four British Orthopteran species has been achieved using artificial neural network analysis [32] and nine species of frogs and three bird species were also identified correctly by using an automated classifier which used linear discriminant analysis as an algorithm [34].

### Acoustic characters

In this study, two sets of song features were used, with five and seven acoustic characters respectively, to examine the influence of number of characters on the percentage of correct classification of thirteen species of field crickets. There was no significant difference in the accuracy of classification when performed using the two character sets. A recent study [34] compared the ability of three machine learning algorithms (linear discriminant analysis, decision tree and support vector machine) to automate the classification of nine frog and three bird species. For this, they proposed two sets of characters based on their recorded calls i.e. 4 and 11 call parameters. The difference between the percentage of correct classification when using 4 and 11 calls in case of both the taxa was <1% indicating no significant statistical effect of higher number of characters. Although the overall difference was found to be small, it was observed that by increasing the number of call parameters from 4 to 11, the accuracy of classification of both taxa increased for all the three algorithms. However, this also reduced the correct classification of a few species by all classification methods. Redundancy in the information content of the acoustic parameters could lead to failure in resolution of classification. In methods such as cluster analysis and DFA, redundancy can also cause misclassification due to increased probability of false clusters along the redundant acoustic parameters. In this study, the choice of two novel acoustic parameters, constancy factor and relative variance was in order to include additional information at the level of the superstructure of song where it exists. However a lack of greater accuracy of classification on using seven characters can be attributed to the fact that calls of only two species out of thirteen had a complex superstructure. The two additional acoustic characters add little information for calls with a simple call structure. As the probability of representation for two calls with superstructures were low in the several randomizations, one could expect little effect of the two additional acoustic features on the resolution of classification. However, when one of these call types was retained in all the randomizations, addition of the two novel acoustic parameters enhanced the accuracy of classification significantly for Coiblemmus sp. but not for Velarifictorus sp.2 (Table 6). We found that though constancy factors were high for both the species with complex calls, relative variance was not very high in case of Velarifictorus sp.2 (Table 1). Moreover the performance of five acoustic characters in resolving the Velarifictorus sp.2 call was higher. These could have led to the failure of the two additional acoustic characters to resolve Velarifictorus sp.2.

In conclusion, both DFA and cluster analysis were effective in correctly identifying species based on their acoustic signals. DFA is the more powerful and accurate method but requires a priori classification of songs and can only be used to identify known song patterns and species. Cluster analysis is less powerful and its accuracy is more contingent on the number of taxa being examined together, but it can be used in situations where the signals of some of the species in the habitat are not previously known, since it does not require a priori grouping of signals or species. Both methods could thus be used to develop quantitative and automated tools for species identification for the cricket fauna in local areas.

## Supporting Information

Figure S1 **Oscillograms and power spectra of the calling songs of** ***Gryllodes sigillatus*** **,** ***Gryllus bimaculatus*** **and** ***Itaropsis*** **subspecies 1.** Scale bar represents 1 second.
(EPS)

Figure S2 **Oscillograms and power spectra of the calling songs of** ***Itaropsis*** **subspecies 2,** ***Itaropsis*** **subspecies 3 and** ***Phonarellus humeralis*** **.** Scale bar represents 1 second.
(EPS)

Figure S3 **Oscillograms and power spectra of the calling songs of** ***Phonarellus minor*** **,** ***Platygryllus*** **sp. and** ***Plebeiogryllus guttiventris*** **.** Scale bar represents 1 second.
(EPS)

Figure S4 **Oscillograms and power spectra of the calling songs of** ***Teleogryllus*** **sp.,** ***Turanogryllus*** **sp. and** ***Velarifictorus*** **sp.1.** Scale bar represents 1 second.
(EPS)

Figure S5 **Oscillograms and power spectra of the calling songs of** ***Velarifictorus*** **sp.2 and** ***Coiblemmus*** **sp.** Scale bar represents 1 second.
(EPS)

Table S1 **Percentage of correctly allocated individuals by discriminant function analysis (DFA) on a dataset with reduced number of individuals per taxon.**
(DOCX)

Table S2 **Percentage of correctly allocated individuals by discriminant function analysis (DFA) for eight taxa with seven acoustic characters with varied amount of misclassification.**
(DOCX)

Table S3 **Results of the Generalised Linear Model analysis with all the pairwise comparisons between taxa5 and the other eight taxa groups and their interactions with character sets for the dataset with fifteen replicates.**
(DOCX)

Table S4 **Results of the Generalised Linear Model analysis with all the pairwise comparisons between taxa5 and the other eight taxa groups and their interactions with character sets for the dataset with five individuals per taxon.**
(DOCX)
