# Supplementary material for: Examining the Effectiveness of Discriminant Function Analysis and Cluster Analysis in Species Identification of Male Field Crickets Based on Their Calling Songs
Source: PLoS One. 2013 Sep 25;8(9):e75930. doi: 10.1371/journal.pone.0075930 (PMC3783383; doi:10.1371/journal.pone.0075930)
Supplement: Table S1 — Percentage of correctly allocated individuals by discriminant function analysis (DFA) on a dataset with reduced number of individuals per taxon. (DOCX) [file pone.0075930.s006.docx]

Table S1. Percentage of correctly allocated individuals by discriminant function analysis (DFA) on a dataset with reduced number of individuals per taxon.

| Number of taxa and characters | 1^st^ randomization | 2^nd^ randomization | 3^rd^ randomization | 4^th^ randomization | 5^th^ randomization | 6^th^ randomization | 7^th^ randomization | 8^th^ randomization | 9^th^ randomization | 10^th^ randomization | Average of correct classification |
| --- | --- | --- | --- | --- | --- | --- | --- | --- | --- | --- | --- |
| 5T:5C | 100 | 100 | 100 | 100 | 100 | 100 | 100 | 100 | 100 | 96 | 100 |
| 5T:7C | 100 | 100 | 100 | 100 | 100 | 100 | 100 | 100 | 100 | 100 | 100 |
| 6T:5C | 90 | 100 | 100 | 100 | 100 | 100 | 100 | 100 | 100 | 100 | 100 |
| 6T:7C | 100 | 100 | 100 | 100 | 100 | 100 | 100 | 100 | 100 | 100 | 100 |
| 7T:5C | 100 | 100 | 100 | 91 | 100 | 100 | 100 | 100 | 100 | 100 | 99 |
| 7T:7C | 100 | 100 | 100 | 100 | 100 | 100 | 100 | 100 | 100 | 100 | 100 |
| 8T:5C | 100 | 93 | 100 | 100 | 100 | 100 | 100 | 100 | 100 | 100 | 99 |
| 8T:7C | 100 | 100 | 100 | 100 | 100 | 100 | 100 | 100 | 100 | 100 | 100 |
| 9T:5C | 93 | 100 | 100 | 100 | 93 | 93 | 100 | 100 | 100 | 100 | 99 |
| 9T:7C | 100 | 100 | 100 | 100 | 100 | 98 | 100 | 100 | 100 | 100 | 100 |
| 10T:5C | 100 | 100 | 100 | 94 | 100 | 100 | 100 | 94 | 100 | 100 | 99 |
| 10T:7C | 100 | 100 | 100 | 98 | 100 | 100 | 100 | 98 | 100 | 100 | 99 |
| 11T:5C | 100 | 95 | 100 | 95 | 95 | 100 | 95 | 95 | 100 | 95 | 97 |
| 11T:7C | 100 | 96 | 100 | 98 | 96 | 100 | 98 | 98 | 100 | 98 | 98 |
| 12T:5C | 100 | 95 | 95 | 95 | 95 | 100 | 95 | 95 | 100 | 94 | 96 |
| 12:7C | 100 | 98 | 98 | 98 | 100 | 100 | 100 | 100 | 100 | 98 | 99 |
| 13T:5C | 95 | 95 | 95 | 95 | 95 | 95 | 95 | 95 | 100 | 100 | 96 |
| 13T:7C | 99 | 99 | 99 | 99 | 99 | 100 | 99 | 99 | 100 | 100 | 99 |
