# Supplementary material for: Examining the Effectiveness of Discriminant Function Analysis and Cluster Analysis in Species Identification of Male Field Crickets Based on Their Calling Songs
Source: PLoS One. 2013 Sep 25;8(9):e75930. doi: 10.1371/journal.pone.0075930 (PMC3783383; doi:10.1371/journal.pone.0075930)
Supplement: Table S2 — Percentage of correctly allocated individuals by discriminant function analysis (DFA) for eight taxa with seven acoustic characters with varied amount of misclassification. (DOCX) [file pone.0075930.s007.docx]

Table S2. Percentage of correctly allocated individuals by discriminant function analysis (DFA) for eight taxa with seven acoustic characters with varied amount of misclassification

| Percentage Misclassification | 1^st^ randomization | 2^nd^ randomization | 3^rd^ randomization | 4^th^ randomization | 5^th^ randomization | 6^th^ randomization | 7^th^ randomization | 8^th^ randomization | 9^th^ randomization | 10^th^ randomization | Average of correct classification |
| --- | --- | --- | --- | --- | --- | --- | --- | --- | --- | --- | --- |
| **5** | 94 | 94 | 94 | 96 | 96 | 96 | 94 | 88 | 94 | 92 | 94 |
| **10** | 90 | 84 | 92 | 86 | 90 | 90 | 91 | 90 | 90 | 92 | 90 |
| **20** | 76 | 80 | 79 | 82 | 79 | 88 | 77 | 80 | 81 | 85 | 80 |
