# Supplementary material for: Examining the Effectiveness of Discriminant Function Analysis and Cluster Analysis in Species Identification of Male Field Crickets Based on Their Calling Songs
Source: PLoS One. 2013 Sep 25;8(9):e75930. doi: 10.1371/journal.pone.0075930 (PMC3783383; doi:10.1371/journal.pone.0075930)
Supplement: Table S4 — Results of the Generalised Linear Model analysis with all the pairwise comparisons between taxa5 and the other eight taxa groups and their interactions with character sets for the dataset with five individuals per taxon. (DOCX) [file pone.0075930.s009.docx]

Table S4. Results of the Generalised Linear Model analysis with all the pairwise comparisons between taxa5 and the other eight taxa groups and their interactions with character sets for the dataset with five individuals per taxon.

| **Categories** | **Estimate** | **Std. Error** | **z value** | **Pr(>\|z\|)** |
| --- | --- | --- | --- | --- |
| (Intercept) | 1.52 | 0.37 | 4.12 | 3.8e-05 |
| taxa6 | -0.02 | 0.5 | -0.05 | 0.96 |
| taxa7 | -0.74 | 0.45 | -1.64 | 0.10 |
| taxa8 | -0.61 | 0.44 | -1.37 | 0.17 |
| taxa9 | -0.62 | 0.44 | -1.41 | 0.16 |
| taxa10 | -0.67 | 0.43 | -1.56 | 0.12 |
| taxa11 | -1.15 | 0.41 | -2.76 | **0.005** |
| taxa12 | -0.93 | 0.41 | -2.25 | **0.02** |
| taxa13 | -0.95 | 0.41 | -2.31 | **0.02** |
| character2 | -0.68 | 0.48 | -1.39 | 0.16 |
| taxa6:character2 | 1.05 | 0.7 | 1.50 | 0.13 |
| taxa7:character2 | 0.8 | 0.61 | 1.33 | 0.18 |
| taxa8:character2 | 0.1 | 0.60 | 1.66 | 0.1 |
| taxa9:character2 | 0.56 | 0.58 | 0.97 | 0.33 |
| taxa10:character2 | 0.35 | 0.57 | 0.63 | 0.53 |
| taxa11:character2 | 0.52 | 0.55 | 0.94 | 0.35 |
| taxa12:character2 | 0.46 | 0.55 | 0.83 | 0.40 |
| taxa13:character2 | 0.32 | 0.54 | 0.58 | 0.56 |
